# A calixpyrrole derivative acts as an antagonist to GPER, a G-protein coupled receptor: mechanisms and models

**DOI:** 10.1242/dmm.021071

**Published:** 2015-10-01

**Authors:** Rosamaria Lappano, Camillo Rosano, Assunta Pisano, Maria Francesca Santolla, Ernestina Marianna De Francesco, Paola De Marco, Vincenza Dolce, Marco Ponassi, Lamberto Felli, Grazia Cafeo, Franz Heinrich Kohnke, Sergio Abonante, Marcello Maggiolini

**Affiliations:** 1 Department of Pharmacy and Health and Nutritional Sciences, University of Calabria, Rende 87036, Italy; 2 U.O.S. Biopolymers and Proteomics, IST-National Institute for Cancer Research, Genova 16132, Italy; 3 Department of Chemical Sciences, University of Messina, Messina 98166, Italy; 4 Breast Cancer Unit, Regional Hospital, Cosenza 87100, Italy

**Keywords:** Breast cancer, Calixpyrroles, Cancer-associated fibroblasts, Estrogen, GPR30/GPER, Signal transduction

## Abstract

Estrogens regulate numerous pathophysiological processes, mainly by binding to and activating estrogen receptor (ER)α and ERβ. Increasing amounts of evidence have recently demonstrated that G-protein coupled receptor 30 (GPR30; also known as GPER) is also involved in diverse biological responses to estrogens both in normal and cancer cells. The classical ER and GPER share several features, including the ability to bind to identical compounds; nevertheless, some ligands exhibit opposed activity through these receptors. It is worth noting that, owing to the availability of selective agonists and antagonists of GPER for research, certain differential roles elicited by GPER compared with ER have been identified. Here, we provide evidence on the molecular mechanisms through which a calixpyrrole derivative acts as a GPER antagonist in different model systems, such as breast tumor cells and cancer-associated fibroblasts (CAFs) obtained from breast cancer patients. Our data might open new perspectives toward the development of a further class of selective GPER ligands in order to better dissect the role exerted by this receptor in different pathophysiological conditions. Moreover, calixpyrrole derivatives could be considered in future anticancer strategies targeting GPER in cancer cells.

## Abstract (precis)

**Drug Discovery Collection:** This paper highlights mechanisms through which a calixpyrrole derivative acts as a newly identified selective antagonist of GPER in different model systems.

## INTRODUCTION

Breast cancer is the most frequent malignancy in women, and mortality of affected individuals is mainly caused by the development of metastatic processes (Siegel et al., 2012), which is driven at least in part by the tumor microenvironment (Schedin and Borges, 2009). Fibroblasts play an essential role in wound healing, regulation of epithelial differentiation and inflammation, and are the predominant cell type in breast tumor stroma (Tomasek et al., 2002). Cancer cells produce secreted factors that activate fibroblasts into proliferative cells, namely cancer-associated fibroblasts (CAFs), which in turn promote the survival and growth of cancer cells (Giannoni et al., 2010; Kalluri and Zeisberg, 2006; Martinez-Outschoorn et al., 2010; Orimo et al., 2005; Polyak and Kalluri, 2010; Tejada et al., 2006). For instance, CAFs elicit an active role in the initiation, progression, metastasis and recurrence of breast tumors (Aboussekhra, 2011).

Estrogens are a group of steroid compounds involved in numerous pathophysiological processes, including in the development of hormone-sensitive tumors (Ascenzi et al., 2006; Yager and Davidson, 2006). In particular, previous studies have supported a reliable association between estrogens and an increased risk of breast cancer (Henderson and Fegelson, 2000; Yue et al., 2013). The mitogenic action of estrogens is mainly mediated by estrogen receptor (ER)α and ERβ, which are ligand-activated transcription factors (O'Malley, 2005; Zhou et al., 2014). In addition, several studies have revealed that a member of the G-protein coupled receptor family, named GPR30 (also known as GPER), is also able to mediate estrogen signaling in diverse types of normal and malignant cells, including breast cancer cells and CAFs derived from breast tumor patients (Madeo and Maggiolini, 2010; Maggiolini and Picard, 2010). Ligand-activated GPER triggers the rapid activation of transduction pathways such as epidermal growth factor receptor (EGFR) and mitogen-activated protein kinases (MAPKs), leading to a specific gene signature and the migration and proliferation of cancer cells and CAFs (Albanito et al., 2007; Lappano et al., 2014; Pandey et al., 2009; Prossnitz and Maggiolini, 2009; Santolla et al., 2012). Of note, GPER expression has been associated with negative clinical features and poor survival rates in patients with hormone-sensitive tumors (Filardo et al., 2006; Smith et al., 2009, 2007; Sjöström et al., 2014), suggesting that GPER might be involved in the stimulatory action exerted by estrogens in these malignancies. Considering that GPER and ER bind promiscuously to many compounds, including endogenous and environmental estrogens as well as antiestrogens (Lappano et al., 2012a; Prossnitz and Barton, 2011), an ongoing major challenge in dissecting the transduction network mediated by GPER is the discovery of novel agents able to act selectively through this receptor, although certain ligands have been identified in our and other previous studies (Bologa et al., 2006; Dennis et al., 2009, 2011; Lappano et al., 2012b; Maggiolini et al., 2015; Sinicropi et al., 2015).

Calixpyrroles are macrocyclic compounds made up of pyrrole units linked by quaternary carbon atoms at their 2,5-positions (Gale et al., 2001). Larger calix[*n*]pyrroles (*n*>4) and hybrid calixpyrroles in which one or more pyrrole units are replaced by a benzo or other heterocyclic unit(s) are also known (Cafeo et al., 2002, 2007). Calixpyrroles have gained considerable interest owing to their ability to bind anions (Gale et al., 1998, 2001), to act as ditopic (ion-pair) receptors (Custelcean et al., 2005) and to host neutral molecules (Allen et al., 1996) that accept NH hydrogen bonds (Gale, 2011). A *meso*-*p*-nitroaniline-calix[4]pyrrole derivative *trans*-coordinated to a platinum(II) [Pt(II)] has been synthesized and for the first time characterized both by structural and *in vitro* analysis as a drug delivery system for *trans*-Pt (Cafeo et al., 2013).

**TRANSLATIONAL IMPACT**

**Clinical issue**

Biological responses to estrogens are mainly mediated by estrogen receptor (ER)α and ERβ, which function as ligand-activated transcription factors. In addition, the G-protein coupled receptor (GPR30/GPER) mediates estrogenic signaling in normal and malignant tissues, including breast cancer cells and cancer-associated fibroblasts (CAFs). Several ER ligands, such as estrogens and ER antagonists, have demonstrated the ability to bind to GPER, eliciting promiscuous and, in certain cases, opposite actions than those elicited via ER binding.

**Results**

In this study, the authors designed and evaluated ‘*in silico*’ diverse calixpyrrole derivatives as potential GPER ligands. In accordance with the results obtained in computational studies, the authors established the molecular mechanisms through which a calixpyrrole derivative, named C4PY, might act as a GPER antagonist in breast tumor cells and CAFs that were obtained from individuals with breast cancer. In particular, they showed that C4PY elicits an inhibitory action on GPER-activated signaling, including the repression of both ERK and Akt phosphorylation, gene transcription, cell proliferation and migration in breast cancer cells and in CAFs. Notably, C4PY is selective for GPER and does not interfere with ER-dependent responses upon estrogen exposure.

**Implications and future directions**

The identification and functional characterization of this novel compound acting as a selective GPER antagonist might represent a valuable tool to further dissect the pharmacology of this receptor and to better differentiate the specific functions elicited by different ER types. In addition, the inhibitory action of C4PY might open new avenues toward innovative pharmacological approaches to target the GPER-mediated stimulatory effects in breast carcinomas. Moreover, this study underlines the fact that strategies against the stimulatory effects exerted by estrogens in ER-negative cancer cells and in key components of the tumor microenvironment (such as CAFs) could be considered as an intriguing opportunity to target breast malignancies.

**Fig. 1.**
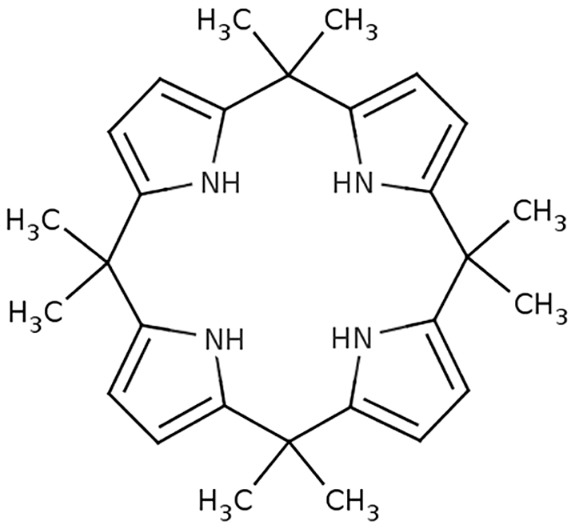
**Chemical structure of meso-octamethylcalix[4]pyrrole (C4PY).**

In order to verify whether similar moieties could be used in medicinal chemistry as protein ligands, we designed and evaluated ‘*in silico*’ diverse calixpyrrole derivatives as suitable GPER ligands. In accordance with the results obtained in computational studies, we ascertained the molecular mechanisms involved in the biological responses to a calix[4]pyrrole derivative [meso-octamethylcalix[4]pyrrole (C4PY)] (Fig. 1), which had the ability to act as a GPER antagonist in breast cancer cells and CAFs used as model systems. Hence, our data suggest that C4PY might be a useful agent toward a better understanding of the role played by GPER in cancer cells as well as in important components of the tumor microenvironment.

## RESULTS

### Molecular modeling and binding assays show that C4PY binds to GPER

**Fig. 2.**
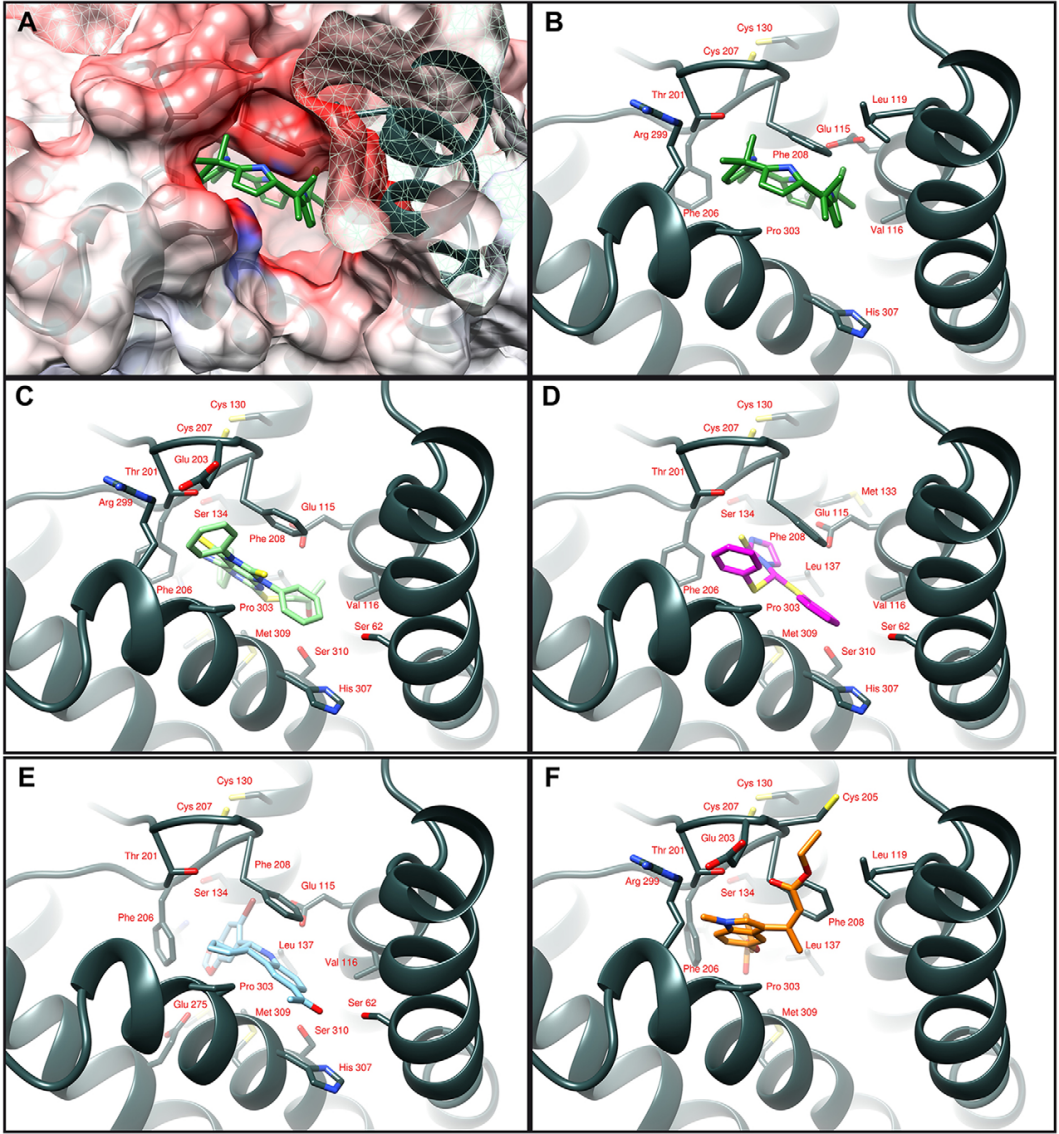
**Ligand binding modes to GPER.** (A) C4PY in the protein binding cleft is drawn in green. The protein surface is colored according to its electrostatic potential (blue positive, red negative). The same ligand binding mode is schematically reported in panel B, where the interacting amino acids are indicated as dark gray sticks. (C,D) The agonists GPER-L1 and GPER-L2 are drawn in light green (C) and purple (D) sticks, respectively. Binding mode of G-1 (cyan) is shown in panel E and the full-antagonist MIBE (orange) in panel F.

**Fig. 3.**
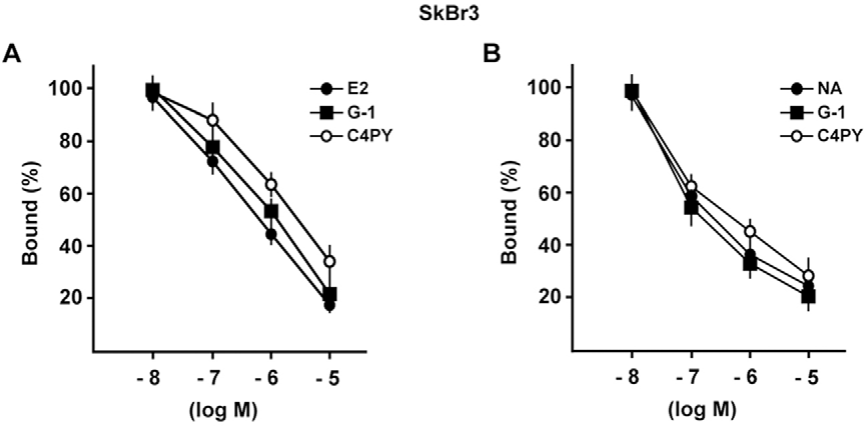
**C4PY is a ligand of GPER.** (A) C4PY competes with [3H]E2 for binding to GPER in SkBr3 cells. Competition curves of increasing concentration of unlabeled E2, G-1 and C4PY expressed as a percentage of maximum specific [3H]E2 binding. Each data point represents the mean±s.d. of triplicate samples of three separate experiments. (B) C4PY competes with [5,6-3H] nicotinic acid (NA) for binding to GPER in SkBr3 cells. Competition curves of increasing concentration of unlabeled NA, G-1 and C4PY expressed as a percentage of maximum specific [5,6-3H] NA binding. Each data point represents the mean±s.d. of three separate experiments performed in triplicate.

We previously identified novel ligands of GPER through a molecular modeling approach in which it was discovered that the ligand binding pocket of GPER consists of a deep cleft in the protein core, contoured by both hydrophobic and hydrophilic amino acids belonging to transmembrane helices (TM) III, TM V, TM VI and TM VII (Lappano et al., 2010, 2012a,b; Rosano et al., 2012). In particular, the three-dimensional model of GPER was successfully tested as a protein target, and docking simulations run *in silico* demonstrated a good affinity of the agonist moiety G-1 for the receptor (Lappano et al., 2010), in accordance with previous data (Bologa et al., 2006). Taking into account the aforementioned findings, we assessed that, among diverse calixpyrroles derivatives, the C4PY binding modes (which describes the orientations of the ligand and receptor, and the conformation of each when they are bound) to GPER are mainly characterized by a network of hydrophobic interactions formed between the macrocycle rings and the protein core residues. This structural characteristic, the dimensions and the conformation adopted meant that C4PY displayed a full interaction with the receptor binding cleft by forming a hydrogen bond with Glu115, different hydrophobic contacts with residues Leu119, Thr201, Phe206, Phe208, Arg299, His302, Pro303 and His307, and then involving amino acids belonging to TM II, EL (extracellular loop) 2 and TM VII (Fig. 2). Table 1 recapitulates the interaction of diverse ligands with the GPER protein residues for a better appraisal of their binding modes. In order to confirm the actual ability of C4PY to bind to GPER, we performed competition assays in ER-negative but GPER-positive SkBr3 breast cancer cells using radiolabeled 17β-estradiol (E2) as a tracer (Lappano et al., 2010). In line with the results obtained in docking simulations, C4PY showed the same capability as E2 and G-1 to displace [3H]E2 (Fig. 3A). In our previous study, nicotinic acid induced stimulatory effects in breast cancer cells and CAFs by binding to GPER and activating the GPER-mediated signaling (Santolla et al., 2014). In order to provide additional evidence on the ligand properties of C4PY to GPER, we performed competition assays using [5,6-3H] nicotinic acid in SkBr3 cells that do not express the nicotinic acid receptors (GPR109A and GPR109B) (Santolla et al., 2014). It is worthy of noting that C4PY displaced the radiolabeled tracer in a dose-dependent manner, as do nicotinic acid and G-1 (Fig. 3B). Collectively, these results demonstrate that C4PY might be considered as a novel ligand of GPER.

**Table 1.**
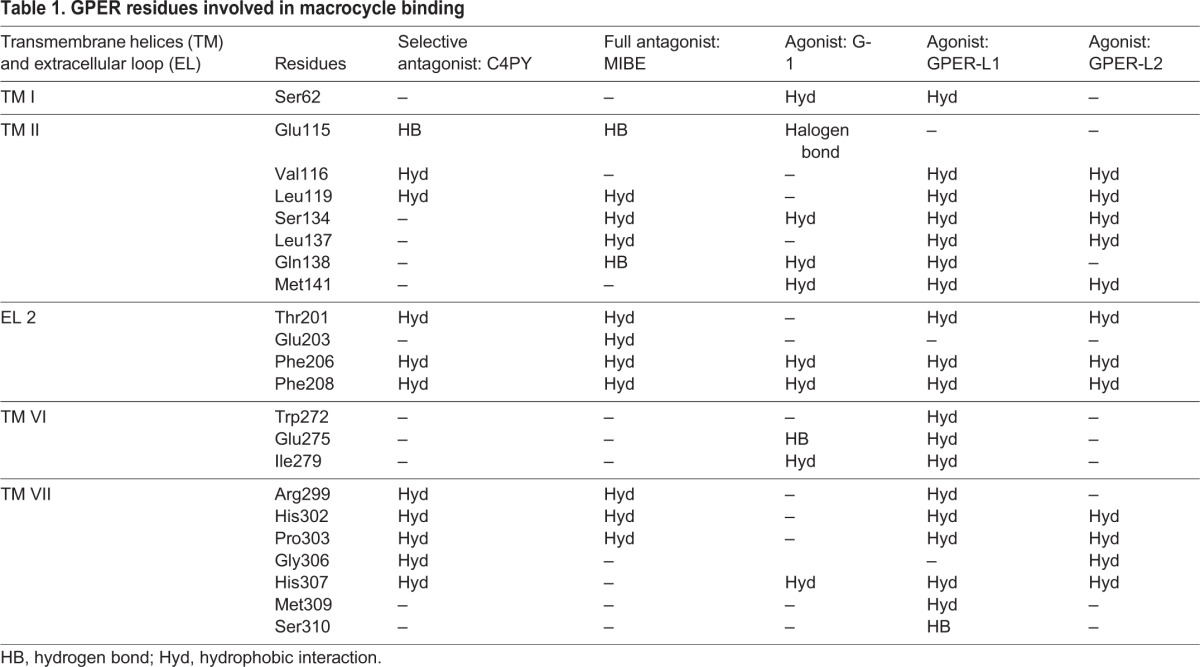
**GPER residues involved in macrocycle binding**

### C4PY acts as a GPER antagonist

**Fig. 4.**
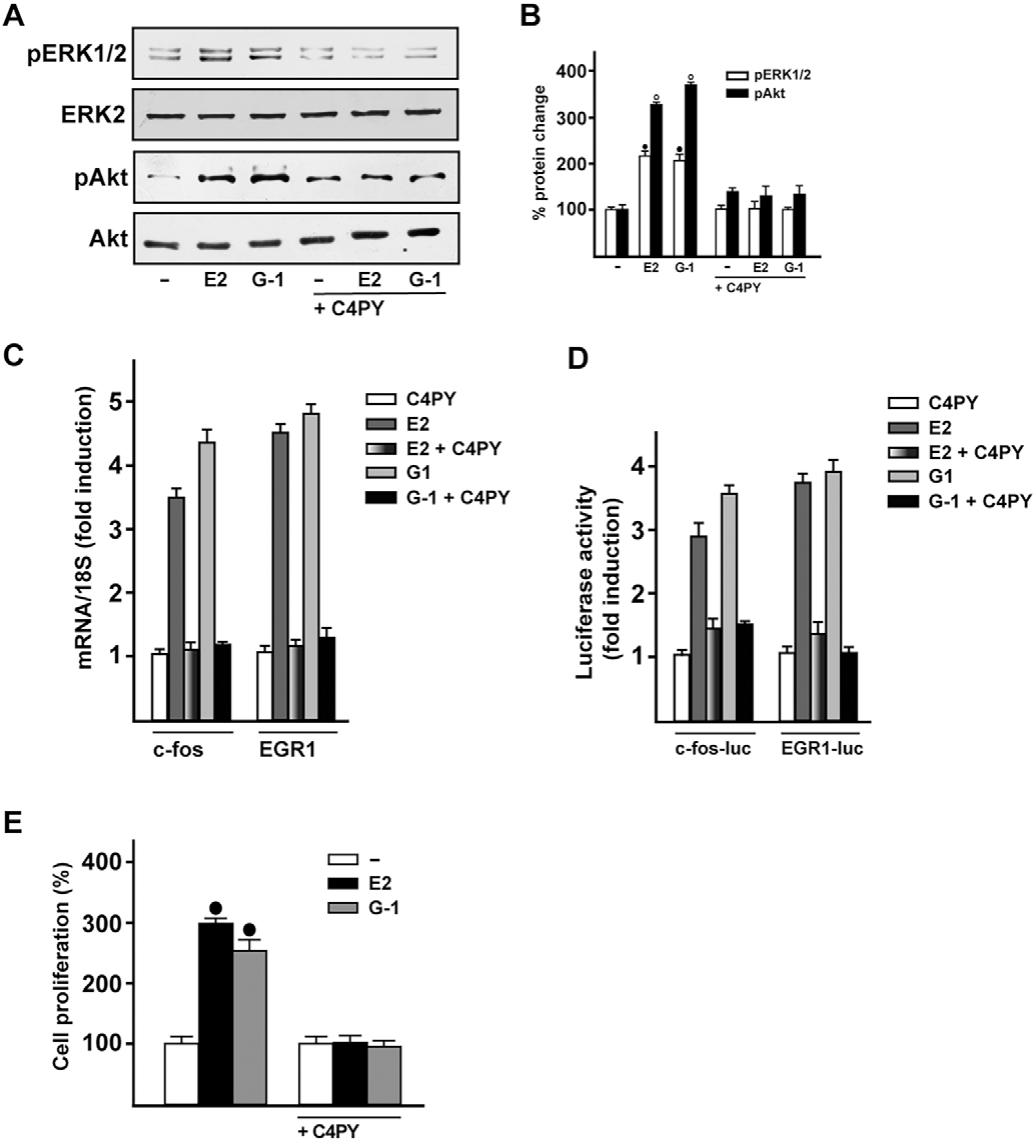
**C4PY exerts inhibitory effects through GPER in SkBr3 breast cancer cells.** (A) ERK1/2 and Akt activation in SkBr3 cells treated for 15 min with 100 nM E2 or 1 µM G-1 is prevented in the presence of 1 µM C4PY. (B) Densitometric analysis of the blots normalized to ERK2 and Akt, respectively. Each data point represents the mean±s.d. of three independent experiments. (C) The mRNA expression of *fos* and *EGR1* induced in SkBr3 cells by 1 h treatment with 100 nM E2 and 1 µM G-1 is inhibited in the presence of 1 µM C4PY, as evaluated by real-time PCR. Results obtained from experiments performed in triplicate were normalized for 18S expression and shown as fold change of RNA expression compared to cells treated with vehicle. Each data point represents the mean±s.d. of three independent experiments performed in triplicate. (D) The transactivation of *fos* and *EGR1* luciferase reporter genes transfected in SkBr3 cells induced by 100 nM E2 and 1 µM G-1 is inhibited by 1 µM C4PY. Luciferase activity was normalized to the internal transfection control *Renilla* luciferase; values are presented as fold change (mean±s.d.) of vehicle control and represent three independent experiments, each performed in triplicate. (E) The proliferation of SkBr3 cells upon treatment with 100 nM E2 and 100 nM G-1 is inhibited by 1 µM C4PY, as indicated. Cells were treated for 5 days with the indicated treatments and counted on day 6. Proliferation of cells receiving vehicle was set as 100%, upon which cell growth induced by treatments was calculated. Each data point is the average ±s.d. of three independent experiments performed in triplicate. (•) indicates *P*<0.05 for cells receiving vehicle (–) versus treatments.

The evaluation of GPCR-mediated signaling includes the early response of the MAPK cascade, which has been used in order to ascertain the potential agonist/antagonist activity of novel drug candidates (May and Hill, 2008). Because ERK phosphorylation indicates the binding of ligand to GPER (Filardo et al., 2000; Maggiolini and Picard, 2010), we aimed to assess the action triggered by C4PY. In SkBr3 cells, C4PY (ranging from 1 nM to 10 µM) did not trigger ERK phosphorylation (data not shown), although it was able to prevent the ERK activation by E2 and G-1 (Fig. 4A,B). Likewise, C4PY inhibited the phosphorylation of Akt induced by both E2 and G-1 (Fig. 4A,B). Considering that the GPER-MAPK-PI3K transduction pathway regulates a number of target genes (Maggiolini et al., 2004; Pandey et al., 2009; Sukhatme et al., 1988; Vivacqua et al., 2012), we then assessed whether the E2- and G-1-induced expression of *fos* and *EGR1* (early growth response protein 1) is repressed by C4PY in SkBr3 cells (Fig. 4C). Further corroborating these findings, C4PY inhibited the transactivation of *fos* and *EGR1* promoter constructs triggered by E2 and G-1 (Fig. 4D). Biologically, we ascertained that the antagonistic action exerted by C4PY through GPER prevents the proliferation of SkBr3 cells that is induced by E2 and G-1 (Fig. 4E).

### C4PY exerts inhibitory effects through GPER in CAFs

**Fig. 5.**
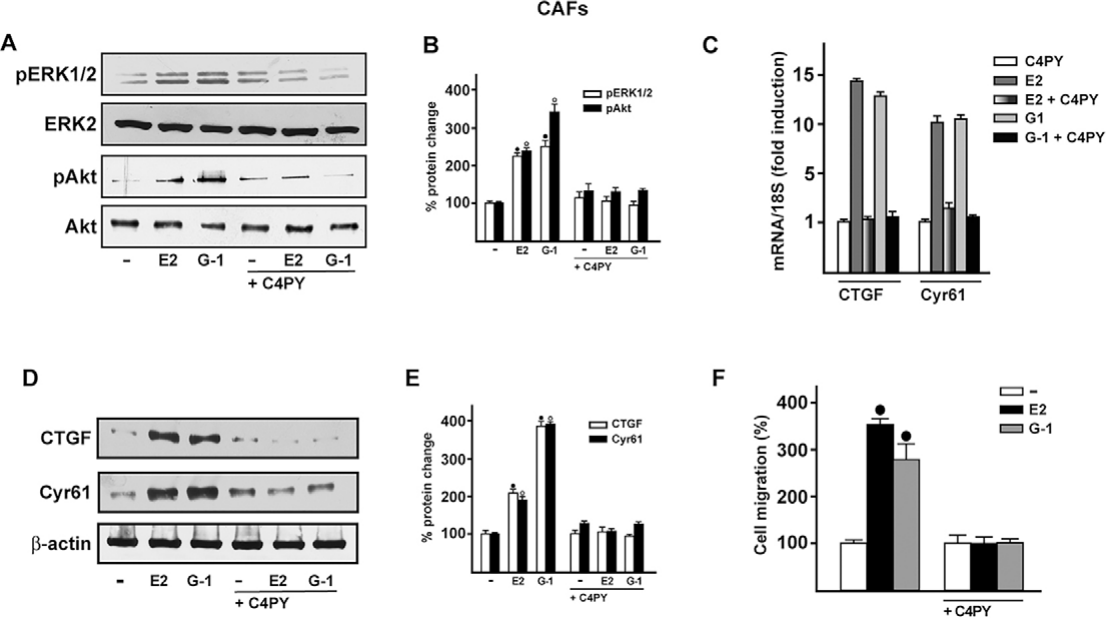
**C4PY exerts inhibitory effects through GPER in CAFs.** (A) ERK1/2 and Akt activation in CAFs treated for 5 min with 1 nM E2 and 100 nM G-1 is prevented by 1 µM C4PY. (B) Densitometric analysis of the blots normalized to ERK2 and Akt, respectively. Each data point represents the mean±s.d. of three independent experiments. (C) The mRNA expression of *CTGF* and *Cyr61* induced in CAFs by 1 h treatment with 1 nM E2 and 100 nM G-1 is prevented by 1 µM C4PY, as evaluated by real-time PCR. Results obtained from experiments performed in triplicate were normalized for 18S expression and shown as fold change of RNA expression compared to cells treated with vehicle. Each data point represents the mean±s.d. of three independent experiments performed in triplicate. (D) CTGF and Cyr61 protein expression induced in CAFs by 2 h treatment with 1 nM E2 and 100 nM G-1 is inhibited in the presence of 1 µM C4PY. (E) Densitometric analyses of the blots normalized to β-actin; values shown represent the mean±s.d. of three independent experiments. (F) The migration of CAFs upon treatment with 1 nM E2 and 100 nM G-1 is inhibited by 1 µM C4PY, as evaluated by Boyden Chamber assay. Each data point is the average ±s.d. of three independent experiments performed in triplicate. (•) and (◦) indicate *P*<0.05 for cells receiving vehicle (–) versus treatments.

**Fig. 6.**
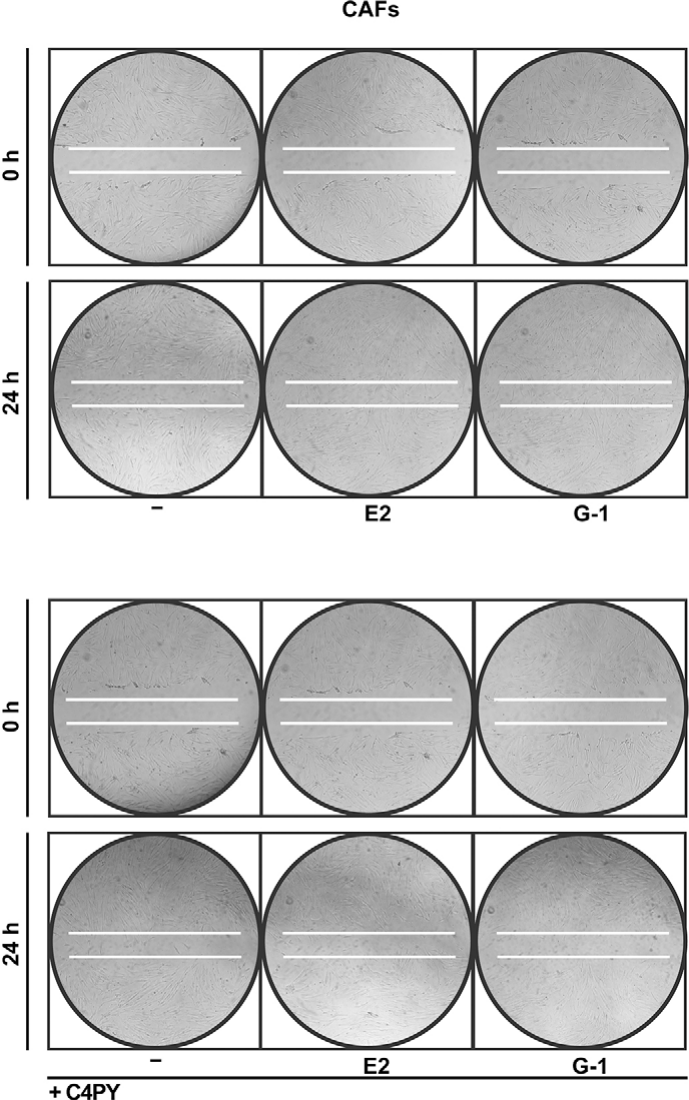
**The migration of CAFs induced by E2 (1 nM) and G-1 (100 nM) is inhibited by 1 µM C4PY, as determined by wound-healing assay.** Data are representative of three independent experiments performed in triplicate.

Increasing amounts of evidence demonstrate that CAFs actively contribute to the growth, expansion and dissemination of breast cancer cells (Al-Ansari et al., 2012; Lebret et al., 2007; Cheng and Weiner, 2003; Gao et al., 2010). Therefore, we investigated whether C4PY elicits an inhibitory action through GPER in CAFs derived from breast cancer patients, because these cells express GPER and lack ER (De Francesco et al., 2014; Madeo and Maggiolini, 2010; Pupo et al., 2013). In accordance with the results obtained in SkBr3 cells, C4PY prevented also in CAFs the rapid ERK and Akt activation induced upon exposure to E2 and G-1 (Fig. 5A,B). Next, we aimed to evaluate the potential of C4PY to alter the expression of two GPER target genes, *CTGF* and *Cyr61* (Pandey et al., 2009), which have been implicated in cell migration (Chen et al., 2007). Notably, the upregulation of CTGF and Cyr61 induced by E2 and G-1 in CAFs at both the mRNA and protein levels was abolished in the presence of C4PY (Fig. 5C-E). As a biological counterpart, the migration of CAFs promoted by both E2 and G-1 was abolished by C4PY (Fig. 5F and Fig. 6), indicating that this compound is able to interfere with relevant responses mediated by GPER also in CAFs that play a stimulatory role within the tumor microenvironment toward cancer progression (Bhowmick et al., 2004).

### C4PY does not interfere with ER-mediated signaling

**Fig. 7.**
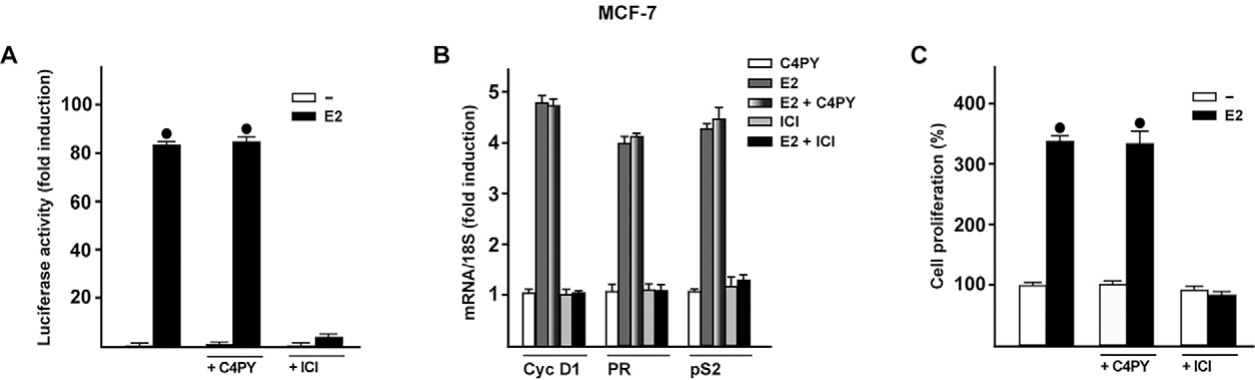
**C4PY does not interfere with the ER-mediated signaling.** (A) MCF-7 cells were transfected with an ER luciferase reporter gene along with the internal transfection control *Renilla* luciferase and then treated with 10 nM E2 in combination with 1 µM C4PY or ICI, as indicated. The normalized luciferase activity values of cells treated with vehicle were set as 1-fold induction, upon which the activity induced by treatments was calculated. Each data point represents the mean±s.d. of three experiments performed in triplicate. (B) The mRNA expression of cyclin D1 (Cyc D1), progesterone receptor (PR) and pS2 induced in MCF-7 cells by 24 h treatment with 10 nM E2 is inhibited by 1 µM ICI, but not by 1 µM C4PY, as evaluated by real-time PCR. Results obtained from experiments performed in triplicate were normalized for 18S expression and shown as fold change of RNA expression compared to cells treated with vehicle. Each data point represents the mean±s.d. of three independent experiments performed in triplicate. (C) The proliferation of MCF-7 cells upon treatment with 10 nM E2 is inhibited by 1 µM ICI, but not by 1 µM C4PY, as indicated. Cells were treated for 5 days with the indicated treatments and counted on day 6. Proliferation of cells receiving vehicle was set as 100%, upon which cell growth induced by treatments was calculated. Each data point is the average ±s.d. of three independent experiments performed in triplicate. (•) indicates *P*<0.05 for cells receiving vehicle (–) versus treatments.

In order to verify whether C4PY might also regulate biological responses mediated by the classical ER, we transiently transfected an ER reporter gene in MCF-7 breast cancer cells. C4PY neither displayed the ability to transactivate ER (data not shown) nor to abrogate the luciferase activity induced by E2 as observed using the ER antagonist ICI (Fig. 7A). In addition, C4PY did not prevent the E2-dependent upregulation of ER target genes such as cyclin D1, progesterone receptor (PR) and pS2, nor the proliferation of MCF-7 cells as shown by ICI (Fig. 7B,C). Together, these data provide evidence that C4PY acts as a selective GPER antagonist in breast cancer cells and CAFs.

## DISCUSSION

In this study, we have identified a novel antagonist ligand of GPER, namely C4PY, which exhibits an inhibitory action on GPER-activated signaling, including the repression of both ERK and Akt phosphorylation, gene transcription, and cell proliferation and migration in breast cancer cells and in CAFs. Of note, C4PY acts selectively through GPER: it does not interfere with the responses triggered by the ER-dependent transduction pathway upon estrogen exposure.

GPCRs constitute a large class of receptors of great biological importance owing to their central role in signal transmission. For instance, abnormal expression, regulation and function of numerous GPCRs have been associated with cancer initiation, progression, invasion and metastasis (Lappano and Maggiolini, 2011, 2012; O'Hayre et al., 2014). Therefore, the pharmacological manipulation of these receptors is very attractive for the development of novel ligands that might become part of innovative strategies targeting tumor development and metastasis. In particular, hormones have been extensively studied among the mitogens that act through GPCRs toward the stimulation of cancer cell growth (Dorsam and Gutkind, 2007). In this regard, it has been demonstrated that the aberrant activity of GPCRs might contribute to the progression of hormone-dependent tumors and their switch into aggressive hormone-insensitive tumors (Dorsam and Gutkind, 2007). In line with these observations, several experimental studies have proved that GPER mediates numerous signaling events in response to estrogens in different types of cancer cells (Prossnitz and Maggiolini, 2009). As it concerns breast malignancy, the role exerted by GPER should be carefully considered owing to its ability to bind not only estrogens but also ER antagonists such as 4-hydroxytamoxifen (OHT) and ICI 182,780, which elicit stimulatory effects particularly in ER-negative cancer cells (Filardo et al., 2000; Lappano et al., 2014; Pandey et al., 2009; Revankar et al., 2005). In addition, GPER signaling is activated by many ER ligands, including natural estrogens and environmental contaminants (Albanito et al., 2015; Maggiolini et al., 2004; Pupo et al., 2012; Thomas and Dong, 2006). It is worthy of noting that we recently identified a compound, named MIBE, that exhibits the peculiar feature of acting as an antagonist ligand of both GPER and ER in breast cancer cells (Lappano et al., 2012a). Overall, the discovery of selective agonist/antagonist ligands of GPER has widely aided research toward the evaluation of the specific activities triggered by GPER in different pathophysiological conditions, including cancer (Bologa et al., 2006; Dennis et al., 2009, 2011; Lappano et al., 2012b; Maggiolini et al., 2015; Prossnitz and Barton, 2011). Moreover, the availability of these ligands has allowed a better understanding of the downstream signaling cascades triggered by GPER, such as the activation of MAPK, PI3-kinase (PI3K) and phospholipase C (PLC), and the increase in cAMP concentrations and intracellular calcium. Of note, GPER mediates the regulation of a distinctive gene signature, which includes transcription factors and cytokines mainly involved in cell survival, proliferation and migration (Lappano et al., 2014; Maggiolini and Picard, 2010; Pandey et al., 2009).

An intricate signaling network has been demonstrated to occur between GPCRs and growth factor receptors (Dorsam and Gutkind, 2007). As it concerns GPER, its physical and functional cross-talk with EGFR has been shown to contribute to the stimulation of diverse types of tumors (Albanito et al., 2008; Filardo et al., 2000; Lappano et al., 2013; Vivacqua et al., 2009). Moreover, the insulin-like growth factor (IGF) system has the ability to regulate the expression and function of GPER in different cancer cells, thus suggesting that GPER might be also engaged by this important growth system toward cancer progression (Bartella et al., 2012; De Marco et al., 2013, 2014, 2015; Lappano et al., 2013). Of note, the mechanisms through which GPER might be involved in the aggressive malignant features were extended to the ability of estrogenic GPER signaling to induce the HIF1α-dependent expression of vascular endothelial growth factor (VEGF) toward breast tumor angiogenesis (De Francesco et al., 2013a,b, 2014; Filice et al., 2009; Recchia et al., 2011). These findings are nicely supported by previous studies reporting that the expression of GPER is correlated with increased tumor size, metastasis and poor outcome in breast cancer (Filardo et al., 2006). The understanding of the overall role exerted by GPER in this neoplasia has become rather complex, considering the strong evidence of its ability to mediate the estrogen stimulation of main components of the tumor microenvironment, such as CAFs (De Francesco et al., 2014; Madeo and Maggiolini, 2010; Pupo et al., 2013, 2014; Vivacqua et al., 2015). Given the established role elicited by CAFs in breast cancer progression, particularly the action at metastatic sites (Aboussekhra, 2011; Kalluri and Zeisberg, 2006), CAFs could be taken into account as promising therapeutic targets in cancer.

Here, we have identified a novel GPER antagonist that could open new avenues toward innovative C4PY-based pharmacological approaches in estrogen-sensitive tumors such as breast carcinomas. In addition, the inhibitory activity exhibited by C4PY in ER-negative breast cancer cells and remarkably in CAFs obtained from patients with breast tumor suggests that novel strategies against both cancer cells and CAFs could improve the therapeutic management of breast malignancies.

## MATERIALS AND METHODS

### Chemical synthesis

The synthesis of meso-octamethylcalix[4]pyrrole (C4PY) has been reported by various authors (Baeyer, 1886; Rothemund and Gage, 1955). In this current work, the procedure was modified as follows. Freshly distilled pyrrole (2 ml, 1.93 g, 0.0288 mol) and an excess of acetone (5 ml, 3.95 g, 0.0681 mol) were diluted in DCM (15 ml) and TFA (2.2 ml, 1.57 g, 0.0137 mol, diluted in 10 ml of DCM) was added under an atmosphere at 0°C in 10 min. The mixture was stirred for 6 h, during which it was allowed to reach room temperature. After the addition of a saturated solution of NaHCO_3_ (to slightly basic pH), the mixture was concentrated under reduced pressure to remove most of the unreacted acetone, and extracted with DCM (3×20 ml). The combined extract were dried (Na_2_SO_4_) and concentrated to give a solid residue, which was crystallized from EtOH to give C4PY (2.31 g, 0.0054 mol, yield 75%, m.p. 275°C dec.), ^1^H-NMR (500 MHz, CD_2_Cl_2_, ppm) δ 7.03 (sbr, 4H, NH), 5.88 and 5.87 (2×s, 2×4H, β-pyrrole CH), 1.49 (s, 24H, CH_3_); ^13^C-NMR (125 MHz, CD_2_Cl_2_, ppm) δ 138.7, 103.0, 35.3, 28.8.

### Molecular modeling and docking simulations

In order to evaluate the potential binding modes of our macrocyclic compounds to GPER, the program GOLD v.5.1 (the Cambridge Crystallographic Data Center, UK) was used in docking simulations. As protein target, the three-dimensional atomic coordinates of the GPER molecular model was utilized in accordance with our previous studies (Lappano et al., 2010). The atom Phe 208 O was considered as ligand binding pocket center, and active site atoms were considered those located within 20 Å from this point (Lappano et al., 2012a). We ran the simulations using the default parameters provided by the software. Residues Tyr123, Gln138, Phe206, Phe208, Glu275, Phe278 and His282 of GPER were defined with flexible side chains, therefore allowing their free rotation. The schematic figures representing protein:ligand complexes were drawn with the program Chimera (Pettersen et al., 2004).

### Reagents

17β-estradiol (E2) was purchased from Sigma-Aldrich Srl (Milan, Italy) and solubilized in ethanol.

G-1 {1-[4-(-6-bromobenzol[1,3]diodo-5-yl)-3a,4,5,9b-tetrahidro3H5cyclopenta[c]quinolin-8yl]-ethanone} was bought from Tocris Bioscience (Bristol, United Kingdom) and dissolved in dimethyl sulfoxide (DMSO). Nicotinic acid (pyridine-3-carboxylic acid) was purchased from Sigma-Aldrich Srl (Milan, Italy) and solubilized in water.

### Cell culture

SkBr3 breast cancer cells were maintained in RPMI 1640 without phenol red supplemented with 10% FBS and 100 mg/ml penicillin/streptomycin (Life Technologies, Milan, Italy). MCF-7 breast cancer cells were maintained in DMEM with phenol red supplemented with 10% FBS and 100 mg/ml penicillin/streptomycin (Life Technologies, Milan, Italy). All cell lines to be processed for immunoblot and RT-PCR assays were switched to medium without serum and phenol red the day before treatments.

CAFs were extracted as previously described (Madeo and Maggiolini, 2010). Briefly, breast cancer specimens were collected from primary tumors of patients who had undergone surgery. Signed informed consent was obtained from all the patients and from the institutional review board(s) of the Regional Hospital of Cosenza, Italy. Tissues from tumors were cut into smaller pieces (1-2 mm diameter), placed in digestion solution (400 IU collagenase, 100 IU hyaluronidase and 10% serum, containing antibiotic and antimycotic solution), and incubated overnight at 37°C. The cells were then separated by differential centrifugation at 90 ***g*** for 2 min. Supernatant containing fibroblasts was centrifuged at 485 ***g*** for 8 min; the pellet obtained was suspended in fibroblasts growth medium (Medium 199 and Ham's F12 mixed 1:1 and supplemented with 10% FBS) and cultured at 37°C in 5% CO_2_. Primary cell cultures of breast fibroblasts were characterized by immunofluorescence. Briefly, cells were incubated with human anti-vimentin (V9) and human anti-cytokeratin 14 (LL001), both from Santa Cruz Biotechnology (DBA, Milan, Italy). To assess fibroblast activation, we used anti-fibroblast activated protein α (FAPα) antibody (H-56; Santa Cruz Biotechnology, DBA, Milan, Italy) (data not shown).

### Plasmids and luciferase assays

The firefly luciferase reporter plasmid for ERα used was XETL (Bunone et al., 1996), which contains the ERE from the *Xenopus* vitellogenin A2 gene (nucleotides −334 to −289), the herpes simplex virus thymidine kinase promoter region (nucleotides −109 to +52), the firefly luciferase coding sequence, and the SV40 splice and polyadenylation sites from plasmid pSV232A/L-AA5. The luciferase reporter plasmid for *fos* encoding a −2.2-kb 5′ upstream fragment of human *fos* was a gift from Dr Kiyoshi Nose (Department of Microbiology, Showa University School of Pharmaceutical Sciences, Hatanodai, Shinagawa-ku, Tokyo, Japan). EGR1-luc plasmid, containing the −600 to +12 5′-flanking sequence from the human *EGR1* gene, was kindly provided by Dr Stephen Safe (Department of Veterinary Physiology and Pharmacology, Texas A&M University, TX, USA). The *Renilla* luciferase expression vector pRL-TK (Promega, Milan, Italy) was used as internal transfection control. Cells were plated into 24-well plates with 500 µl of regular growth medium/well the day before transfection. For the transfection of the ER reporter gene in MCF-7 cells, standard medium was replaced with medium supplemented with 1% charcoal-stripped (CS) FBS lacking phenol red and serum on the day of transfection, which was performed by using X-tremeGENE 9 DNA Transfection Reagent as recommended by the manufacturer (Roche Molecular Biochemicals, Milan, Italy) with a mixture containing 0.5 µg of reporter plasmid and 2 ng of pRL-TK. After 6 h, the medium was replaced again with serum-free medium lacking phenol red and supplemented with 1% CS-FBS; treatments were added at this point and cells were incubated for an additional 18 h. For the luciferase assays of the *fos* and *EGR1* reporter plasmids, on the day of transfection, SkBr3 cell medium was replaced with RPMI without phenol red and serum, and transfection was performed using X-tremeGENE 9 DNA Transfection Reagent (Roche Molecular Biochemicals, Milan, Italy) and a mixture containing 0.5 μg of each reporter plasmid and 5 ng of pRL-TK. After 6 h, treatments were added and cells were incubated for 18 h. Luciferase activity was then measured using the Dual Luciferase Kit (Promega, Milan, Italy) according to the manufacturer's recommendations. Firefly luciferase activity was normalized to the internal transfection control provided by the *Renilla* luciferase activity. The normalized relative light unit values obtained from cells treated with vehicle were set as 1-fold induction, upon which the activity induced by treatments was calculated.

### Ligand binding assays

In ligand binding assays, SkBr3 cells were grown in 10-cm cell-culture dishes, washed two times and incubated either with 1 nM [2,4,6,7-3H]E2 (89 Ci/mmol; Amersham Bioscience, GE Healthcare, Milan, Italy) or with 50 nM [5,6-3H] nicotinic acid (50-60 Ci/mmol; BIOTREND, Chemikalien GmbH, Köln, Germany) in the presence or absence of increasing concentrations of nonlabeled competitors, as indicated. Then, cells were incubated for 2 h at 37°C and washed three times with ice-cold PBS; the radioactivity collected by 100% ethanol extraction was measured by liquid scintillation counting. Competitor binding was expressed as a percentage of maximal specific binding.

### Gene expression studies

Total RNA was extracted and cDNA was synthesized by reverse transcription as previously described (Lappano et al., 2011). The expression of selected genes was quantified by real-time PCR using Step One sequence detection system (Applied Biosystems Inc., Milan, Italy). Gene-specific primers were designed using Primer Express version 2.0 software (Applied Biosystems Inc., Milan, Italy). For cyclin D1, *PR*, *pS2*, *fos*, *CTGF*, *Cyr61*, *EGR1* and the ribosomal protein 18S, which was used as a control gene to obtain normalized values, the primers were: 5′-GTCTGTGCATTTCTGGTTGCA-3′ (cyclin D1 forward) and 5′-GCTGGAAACATGCCGGTTA-3′ (cyclin D1 reverse); 5′-GAGTTGTGAGAGCACTGGATGCT-3′ (PR forward) and 5′-CAACTGTATGTCTTGACCTGGTGAA-3′ (PR reverse); 5′-GCCCCCCGTGAAAGAC-3′ (pS2 forward) and 5′-CGTCGAAACAGCAGCCCTTA-3′ (pS2 reverse); 5′-CGAGCCCTTTGATGACTTCCT-3′ (fos forward) and 5′-GGAGCGGGCTGTCTCAGA-3′ (fos reverse); 5′-ACCTGTGGGATGGGCATCT-3′ (CTGF forward) and 5′-CAGGCGGCTCTGCTTCTCTA-3′ (CTGF reverse); 5′-GAGTGGGTCTGTGACGAGGAT-3′ (Cyr61 forward) and 5′-GGTTGTATAGGATGCGAGGCT-3′ (Cyr61 reverse); 5′-GCCTGCGACATCTGTGGAA-3′ (EGR1 forward) and 5′-CGCAAGTGGATCTTGGTATGC-3′ (EGR1 reverse); and 5′-GGCGTCCCCCAACTTCTTA-3′ (18S forward) and 5′-GGGCATCACAGACCTGTTATT-3′ (18S reverse), respectively.

### Western blotting

Cells were grown in 10-cm dishes, exposed to treatments, and then lysed in 500 μl of 50 mmol/l NaCl, 1.5 mmol/l MgCl_2_, 1 mmol/l EGTA, 10% glycerol, 1% Triton X-100, 1% sodium dodecyl sulfate (SDS), and a mixture of protease inhibitors containing 1 mmol/l aprotinin, 20 mmol/l phenylmethylsulfonyl fluoride and 200 mmol/l sodium orthovanadate. Protein concentration was determined using Bradford reagent according to the manufacturer's recommendations (Sigma-Aldrich, Milan, Italy). Equal amounts of whole protein extract were resolved on a 10% SDS-polyacrylamide gel, transferred to a nitrocellulose membrane (Amersham Biosciences, GE Healthcare, Milan, Italy), probed overnight at 4°C with antibodies against CTGF (L-20), CYR61 (H-78), β-actin (C-2), phosphorylated AKT 1/2/3 (Ser 473), AKT1/2/3 (H-136), phosphorylated ERK1/2 (E-4) and ERK2 (C-14) (all purchased from Santa Cruz Biotechnology, DBA, Milan, Italy), and then revealed using the ECL™ Western Blotting Analysis System (GE Healthcare, Milan, Italy).

### Proliferation assay

For quantitative proliferation assays, cells (1×10^5^) were seeded in 24-well plates in regular growth medium. Cells were washed once they had attached and then incubated in medium containing 2.5% charcoal-stripped FBS with the indicated treatments; medium was renewed every 2 days (with treatments) before counting using the Countess Automated Cell Counter, as recommended by the manufacturer's protocol (Life Technologies, Milan, Italy).

### Migration assays

Migration assays were performed with CAFs in triplicate using Boyden chambers (Costar Transwell, 8 mm polycarbonate membrane, Sigma-Aldrich, Milan, Italy). CAFs were trypsinized and seeded in the upper chambers. Treatments were added to the medium without serum in the bottom wells where applicable. At 6 h after seeding, cells on the bottom side of the membrane were fixed and counted. Moreover, wound-healing assays were also performed in order to visualize cell migration. Cells (1×10^6^/well) were seeded onto six-well plates in regular medium. After 18 h, wounds were created by dragging a 200-μl pipette tip through the cell monolayer; the medium was replaced with 2.5% charcoal-stripped FBS and the treatments were added. Cells were allowed to migrate for 24 h; the gap area was then photographed and migration distances were measured.

### Statistical analysis

Statistical analysis was done using ANOVA followed by Newman-Keuls’ testing to determine differences in means. *P*<0.05 was considered as statistically significant.

## Notes

This article is part of a subject collection on Model Systems in Drug Discovery: from Bench to Patient. See related articles in this collection at http://dmm.biologists.org/cgi/collection/drugdiscovery
